# Supplementary material for: Bronchial epithelial transcriptomics and experimental validation reveal asthma severity-related neutrophilc signatures and potential treatments
Source: Commun Biol. 2024 Feb 14;7:181. doi: 10.1038/s42003-024-05837-y (PMC10864370; doi:10.1038/s42003-024-05837-y)
Supplement: Supplementary file 2 — Supplementary Tables [file 42003_2024_5837_MOESM2_ESM.pdf]

Supplementary Table 1. Clinical information of the recruited patients in GSE89809 dataset.

Variable	Mild asthma (n=14)	Moderate asthma (n=13)	Severe asthma (n=11)	Healthy (n=18)
Gender(M/F)	8/6	6/7	5/6	12/8
Age (mean [range]), year	30 (21-64)	35.07 (21-56)	53 (31-67)	33.9 (20-65)
Smoking status				
Never	13	12	6	15
Former	1	1	4	3
Current	0	0	1	0
Asthma control questionnaire	0.87 (0-2.14)	1.26 (0-2.57)	2.66 (1.14-3.57)	0
Allergic rhinitis				
Yes	11	7	5	0
No	3	6	6	18
Nasal polyps				
Yes	0	1	4	0
No	14	12	7	18
Inhaled corticosteroid (ICS) dose	14.29 (0-200)	676.92 (200-2000)	1618.18 (400-3400)	0
Oral corticosteroid dose	0 (0-0)	0 (0-0)	2.73 (0-15)	0
FEV1	3.43 (2.1-4.1)	3.47 (2.2-4.67)	4.02 (1.1-2.5)	4.02 (2.5-5.6)
FEV1 Reversibility	13.65 (4-23)	13.8 (2.2-28)	5.13 (0-51.4)	5.13 (-1.1-14)
FVC	4.58 (3.2-6)	4.5 (3-6.4)	4.94 (1.8-4.1)	4.94 (3.14-6.1)
GINA level of control, n				
Controlled	7	2	0	0
Partly Controlled	6	9	0	0
Uncontrolled	1	2	11	0

FEV1: forced expiratory volume in the first second; FVC: forced vital capacity; GINA: Global Initiative for Asthma.

Supplementary Table 2. Primer sequences for RT-qPCR in vivo research.

Gene	Primer	Sequence (5’-3’)
ACTIN	Forward prime	ACTGTCGAGTCGCGTCC
	Reverse prime	CTGACCCATTCCCACCATCA
PTPRC	Forward prime	ATGGTCCTCTGAATAAAGCCCA
	Reverse prime	TCAGCACTATTGGTAGGCTCC
TLR2	Forward prime	GCAAACGCTGTTCTGCTCAG
	Reverse prime	AGGCGTCTCCCTCTATTGTATT
MMP9	Forward prime	CCATGTCACTTTCCCTTCACCTT
	Reverse prime	TCTCACTAGGGCAGAAACCAAAT
TYROBP	Forward prime	GAGTGACACTTTCCCAAGATGC
	Reverse prime	CCTTGACCTCGGGAGACCA
CXCR1	Forward prime	TCTGGACTAATCCTGAGGGTG
	Reverse prime	GCCTGTTGGTTATTGGAACTCTC
FCGR3B	Forward prime	CATCCAAAGTGCCAGAGTTAAGG
	Reverse prime	TGCCGTTCTGTAAATAGGTGACC
FPR1	Forward prime	CATTTGGTTGGTTCATGTGCAA
	Reverse prime	AATACAGCGGTCCAGTGCAAT
CCR1	Forward prime	CTCATGCAGCATAGGAGGCTT
	Reverse prime	ACATGGCATCACCAAAAATCCA
CXCR2	Forward prime	ATGCCCTCTATTCTGCCAGAT
	Reverse prime	GTGCTCCGGTTGTATAAGATGAC

Supplementary Table 3. Primer sequences for RT-qPCR in vitro research.

Gene	Primer	Sequence (5’-3’)
ACTIN	Forward prime	CACCCAGCACAATGAAGATCAAG
	Reverse prime	TCATAGTCCGCCTAGAAGCATTT
PTPRC	Forward prime	TCCTGCAGAACCCAAGGAATTAA
	Reverse prime	TACCTCTTCTGTTTCCGCACTTT
TLR2	Forward prime	AAGCACTGGACAATGCCACA
	Reverse prime	ACCATTGCGGTCACAAGACA
MMP9	Forward prime	GAGATGCGTGGAGAGTCGAAATC
	Reverse prime	CAATAGGTGATGTTGTGGTGGTG
TYROBP	Forward prime	GGTGCTGACAGTGCTCATTGC
	Reverse prime	GGCGACTCGGTCTCAGTGATAC
CXCR1	Forward prime	GATTCCTCAAGATCCTGGCTATGC
	Reverse prime	AGAGACATTGACAGACGAAGAAGTG
S100A12	Forward prime	GCATCTGGAGGGAATTGTCAATATC
	Reverse prime	TGGCTACCAGGGATATGAATTCT
FPR1	Forward prime	GACCCAGACCTAGAACTACCCA
	Reverse prime	CAGAGATGTTCGTGGGGAGAG
CCR1	Forward prime	ATGACACGACCACAGAGTTTGAC
	Reverse prime	GGACCAGGACCACCAGGATG
CXCR2	Forward prime	GCATCAGTGTGGACCGTTACC
	Reverse prime	GCCAGGAGCAAGGACAGACC
FCGR3B	Forward prime	ATCTTCAAGCAGGGAAGCCC
	Reverse prime	TGTTGCTTTGCTGTGAGGGA

Supplementary Table 4: Results of biological process enrichment analysis of 46 genes.

ID	Description	*p*. adjust	Gene symbols	Counts
GO:0030595	Leukocyte chemotaxis	2.17E-11	CXCR2/CXCR1/CCL26/CCR7/CSF3R/CCR1/FPR2/ CXCR4/S100A12/PPBP/CXCL5/S100A9/CXCL3	13
GO:0030593	Neutrophil chemotaxis	6.60E-11	CXCR2/CXCR1/CCL26/CCR7/CSF3R/S100A12/ PPBP/CXCL5/S100A9/CXCL3	10
GO:0060326	Cell chemotaxis	1.33E-10	CXCR2/CXCR1/CCL26/CCR7/CSF3R/CCR1/FPR2/ CXCR4/S100A12/PPBP/CXCL5/S100A9/CXCL3	13
GO:0019221	Cytokine-mediated signaling pathway	1.33E-10	CXCR2/CXCR1/CCL26/CCR7/TNFRSF1B/CSF3R/ IL1R2/CCR1/IL2RG/PTPRC/CXCR4/PPBP/ CSF2RB/CXCL5/CXCL3	15
GO:0097529	Myeloid leukocyte migration	1.33E-10	CXCR2/CXCR1/CCL26/CCR7/CSF3R/CCR1/ FPR2/S100A12/PPBP/CXCL5/S100A9/CXCL3	12
GO:1990266	Neutrophil migration	1.37E-10	CXCR2/CXCR1/CCL26/CCR7/CSF3R/S100A12/ PPBP/CXCL5/S100A9/CXCL3	10
GO:0071621	Granulocyte chemotaxis	1.48E-10	CXCR2/CXCR1/CCL26/CCR7/CSF3R/ S100A12/PPBP/CXCL5/S100A9/CXCL3	10
GO:0070098	Chemokine-mediated signaling pathway	1.89E-10	CXCR2/CXCR1/CCL26/CCR7/CCR1/ CXCR4/PPBP/CXCL5/CXCL3	9
GO:1990868	Response to chemokine	3.30E-10	CXCR2/CXCR1/CCL26/CCR7/CCR1/ CXCR4/PPBP/CXCL5/CXCL3	9
GO:1990869	Cellular response to chemokine	3.30E-10	CXCR2/CXCR1/CCL26/CCR7/CCR1/ CXCR4/PPBP/CXCL5/CXCL3	9

Supplementary Table 5: The Kyoto Encyclopedia of Genes and Genomes enrichment analysis of 46 genes.

ID	Description	p.adjust	geneID	Count
hsa04060	Cytokine-cytokine receptor interaction	1.70E-11	CXCR2/CXCR1/CCL26/CCR7/TNFRSF1B/CSF3R/IL1R2/CCR1/LTB/IL2RG/CXCR4/PPBP/CSF2RB/CXCL5/CXCL3/INHBA	16
hsa04061	Viral protein interaction with cytokine and cytokine receptor	4.32E-11	CXCR2/CXCR1/CCL26/CCR7/TNFRSF1B/CCR1/IL2RG/CXCR4/PPBP/CXCL5/CXCL3	11
hsa04062	Chemokine signaling pathway	5.95E-07	CXCR2/CXCR1/CCL26/CCR7/CCR1/PIK3R5/CXCR4/PPBP/CXCL5/CXCL3	10
hsa05323	Rheumatoid arthritis	1.27E-04	HLA-DQB1/HLA-DQA1/LTB/TLR2/CXCL5/CXCL3	6
hsa04145	Phagosome	1.45E-04	HLA-DQB1/FCGR3B/HLA-DQA1/MARCO/TLR2/TFRC/MSR1	7
hsa05150	Staphylococcus aureus infection	1.56E-03	HLA-DQB1/FCGR3B/FPR1/HLA-DQA1/FPR2	5
hsa04640	Hematopoietic cell lineage	1.56E-03	HLA-DQB1/CSF3R/IL1R2/HLA-DQA1/TFRC	5
hsa05145	Toxoplasmosis	2.34E-03	HLA-DQB1/HSPA6/HLA-DQA1/PIK3R5/TLR2	5
hsa05321	Inflammatory bowel disease	2.96E-03	HLA-DQB1/HLA-DQA1/IL2RG/TLR2	4
hsa04612	Antigen processing and presentation	4.65E-03	HLA-DQB1/HSPA6/HLA-DQA1/IFI30	4
hsa05140	Leishmaniasis	4.65E-03	HLA-DQB1/FCGR3B/HLA-DQA1/TLR2	4
hsa04657	IL-17 signaling pathway	9.05E-03	CXCL5/S100A9/CXCL3/MMP9	4
hsa04144	Endocytosis	9.34E-03	CXCR2/CXCR1/HSPA6/IL2RG/CXCR4/TFRC	6
hsa04672	Intestinal immune network for IgA production	1.22E-02	HLA-DQB1/HLA-DQA1/CXCR4	3
hsa04613	Neutrophil extracellular trap formation	1.38E-02	FCGR3B/FPR1/FPR2/TLR2/AQP9	5
hsa04668	TNF signaling pathway	1.38E-02	TNFRSF1B/CXCL5/CXCL3/MMP9	4

hsa05134	Legionellosis	1.48E-02	HSPA6/TLR2/CXCL3	3
hsa05120	Epithelial cell signaling in Helicobacter pylori infection	2.64E-02	CXCR2/CXCR1/CXCL3	3
hsa04658	Th1 and Th2 cell differentiation	5.06E-02	HLA-DQB1/HLA-DQA1/IL2RG	3
hsa05310	Asthma	5.06E-02	HLA-DQB1/HLA-DQA1	2

Supplementary Table 6. Identified genes and transcription factors.

Identified genes	Transcription factors
FCGR3B	POLR2A, ZNF407, CDC5L, IRF1, ID3, MEF2D, ATF1, CREB3, ZBTB40, ARNT, NR4A1, PTRF, ARID1B, IKZF1, DNMT1, ZNF76, MTA2, NFRKB
FPR1	NFRKB, ARID1B
MMP9	ID3, CTCF, MXD3, RFXANK, MXD4, CUX1, ZBTB26, SSRP1, KDM5A, EZH2, GATAD1, CTBP2, ZNF7, MAZ, ZKSCAN1, KDM1A, MTA1, KLF16, DRAP1, TFAP4, HHEX, ZNF341
PTPRC	IRF1, NFRKB, SPI1, POLR2H, ZNF589, GABPA, TCF7, CBFB, GTF2E2, HMBOX1, CEBPD, NFIC, FOXA2, RUNX3, ZNF639, JUNB, TEAD4, MYNN, ELK1, ZNF175, MLLT1
TYROBP	ATF1, ARID1B, ZNF76, MTA2, CBFB, MYNN, ZNF24, FOXM1, ZNF101, KLF7, HDGF, CHD4, ZNF641, ZNF264, ZNF121, ZNF366, ZBTB1, ZNF423, TBX21, ZNF239, KLF9, NR2C2, ZNF501, ZNF547, TSHZ1, GFI1B, ZNF585B, IRF4, BCL11A, ZBTB33, ZNF610, ZNF394, ZFP37, HIC1, BCL11B, ZBTB7A, EBF1, ZBTB17, ELF1, ZNF18, HMGN3, ZNF623, ZNF146, TARDBP, INSM2, EGR1, ZEB1, ZNF8, TRIM22, KLF8
S100A12	FOS, CEBPG, STAT3
TLR2	STAT3

Supplementary Table 7. Drug-gene interactions in asthma.

Enrichment FDR	Pathway Genes	Fold Enrichment	Pathway	Genes
7.57E-07	7	1085.52	2-(4-isobutylphenyl) propionylmethane sulfonamide (Reperixin)	MMP9/CXCR1/CXCR2
2.53E-05	123	82.37	Lipopolysaccharide E. coli O26-B6	MMP9/TLR2/CXCR1/CXCR2
6.55E-05	39	194.84	Cytochalasin D	MMP9/CXCR1/CXCR2
9.52E-05	1863	9.52	Methotrexate	TYROBP/MMP9/TLR2/FCGR3B/S100A12/FPR1/CXCR2
1.01E-04	5	1013.16	SB 225002	MMP9/CXCR2
1.01E-04	5	1013.16	Oleandrin	CXCR1/CXCR2
1.01E-04	579	21.87	Tetradecanoylphorbol Acetate	TYROBP/MMP9/TLR2/CXCR1/CXCR2
1.32E-04	6	844.30	BIRB 796	CXCR1/CXCR2
2.89E-04	777	16.30	Lipopolysaccharides	MMP9/TLR2/CXCR1/CCR1/CXCR2
3.17E-04	10	506.58	Lipoteichoic acid	TLR2/CXCR2

FDR: false discovery rate.
