# Supplementary material for: Bronchial epithelial transcriptomics and experimental validation reveal asthma severity-related neutrophilc signatures and potential treatments
Source: Commun Biol. 2024 Feb 14;7:181. doi: 10.1038/s42003-024-05837-y (PMC10864370; doi:10.1038/s42003-024-05837-y)
Supplement: Supplementary file 3 — Description of Additional Supplementary Files [file 42003_2024_5837_MOESM3_ESM.pdf]

Description of Additional Supplementary Files

File name: Supplementary Data 1
Description: The numerical source data underlying all graphs.
