# Supplementary material for: Bronchial epithelial transcriptomics and experimental validation reveal asthma severity-related neutrophilc signatures and potential treatments
Source: Commun Biol. 2024 Feb 14;7:181. doi: 10.1038/s42003-024-05837-y (PMC10864370; doi:10.1038/s42003-024-05837-y)
Supplement: Supplementary file 5 — Reporting Summary [file 42003_2024_5837_MOESM5_ESM.pdf]

nature portfolio

Corresponding author(s): COMMSBIO-23-1304

Last updated by author(s): Dec 1, 2023

Reporting Summary

Nature Portfolio wishes to improve the reproducibility of the work that we publish. This form provides structure for consistency and transparency in reporting. For further information on Nature Portfolio policies, see our Editorial Policies and the Editorial Policy Checklist.

Statistics

For all statistical analyses, confirm that the following items are present in the figure legend, table legend, main text, or Methods section.

n/a	Confirmed	
☐	☒	The exact sample size (n) for each experimental group/condition, given as a discrete number and unit of measurement
☐	☒	A statement on whether measurements were taken from distinct samples or whether the same sample was measured repeatedly
☐	☒	The statistical test(s) used AND whether they are one- or two-sided<br>*Only common tests should be described solely by name; describe more complex techniques in the Methods section.*
☐	☒	A description of all covariates tested
☐	☒	A description of any assumptions or corrections, such as tests of normality and adjustment for multiple comparisons
☐	☒	A full description of the statistical parameters including central tendency (e.g. means) or other basic estimates (e.g. regression coefficient) AND variation (e.g. standard deviation) or associated estimates of uncertainty (e.g. confidence intervals)
☐	☒	For null hypothesis testing, the test statistic (e.g. F, t, r) with confidence intervals, effect sizes, degrees of freedom and P value noted<br>*Give P values as exact values whenever suitable.*
☒	☐	For Bayesian analysis, information on the choice of priors and Markov chain Monte Carlo settings
☒	☐	For hierarchical and complex designs, identification of the appropriate level for tests and full reporting of outcomes
☒	☐	Estimates of effect sizes (e.g. Cohen's d, Pearson's r), indicating how they were calculated

Our web collection on statistics for biologists contains articles on many of the points above.

Software and code

Policy information about availability of computer code

Data collection	The data is available at the Gene Expression Omnibus public repository.
Data analysis	The data and R code underlying this article is available at 4TU.RearchData (DOI: https://doi.org/10.4121/19537258.v1) and the Gene Expression Omnibus public repository.

For manuscripts utilizing custom algorithms or software that are central to the research but not yet described in published literature, software must be made available to editors and reviewers. We strongly encourage code deposition in a community repository (e.g. GitHub). See the Nature Portfolio guidelines for submitting code & software for further information.

Data

Policy information about availability of data

All manuscripts must include a data availability statement. This statement should provide the following information, where applicable:

- Accession codes, unique identifiers, or web links for publicly available datasets
- A description of any restrictions on data availability
- For clinical datasets or third party data, please ensure that the statement adheres to our policy

The data and R code underlying this article is available at 4TU.RearchData (DOI: https://doi.org/10.4121/19537258.v1) and the Gene Expression Omnibus public repository.

Research involving human participants, their data, or biological material

Policy information about studies with human participants or human data. See also policy information about sex, gender (identity/presentation), and sexual orientation and race, ethnicity and racism.

Reporting on sex and gender	In our clinical study, we used public databases to obtain clinical samples for analysis. Gender was not a factor that we specifically considered in relation to the disease being studied. As such, we did not actively control for it during the selection of clinical samples. When constructing an animal model of asthma, however, we chose to use female mice due to their greater susceptibility to developing the condition. This decision was based on previous research indicating that female mice are more easily induced to develop asthma models than male mice. By using female mice, we aimed to increase the reliability and reproducibility of our results, while also minimizing the number of animals used in our experiment.
Reporting on race, ethnicity, or other socially relevant groupings	When conducting research studies, it is often necessary to establish inclusion and exclusion criteria for both the data set and the study population. Inclusion criteria are used to identify and select the specific data or individuals that meet the research objectives, while exclusion criteria are used to exclude those who do not meet the specific requirements or have certain characteristics that may affect the validity of the study. In our study, we chose to establish inclusion criteria for obtaining the data set. However, we did not establish any exclusion criteria for the study population.
Population characteristics	see above
Recruitment	The selection criteria for the dataset were set as follows: (a) the dataset must contain mild, moderate, and severe asthma patients; (b) the samples in the dataset must derive from airway epithelium.
Ethics oversight	As the clinical samples used in our study were obtained from a public database, which likely contains anonymized or de-identified data, it is likely that no ethical considerations arise from their use.

Note that full information on the approval of the study protocol must also be provided in the manuscript.

Field-specific reporting

Please select the one below that is the best fit for your research. If you are not sure, read the appropriate sections before making your selection.

☒ Life sciences ☐ Behavioural & social sciences ☐ Ecological, evolutionary & environmental sciences

For a reference copy of the document with all sections, see nature.com/documents/nr-reporting-summary-flat.pdf

Life sciences study design

All studies must disclose on these points even when the disclosure is negative.

Sample size	We followed the 3R principle and selected a limited number of mice per group in animal model.
Data exclusions	We selected a gene expression dataset from the Gene Expression Omnibus (GEO, https://www.ncbi.nlm.nih.gov/geo/) database of the National Center for Biotechnology Information. The selection criteria for the dataset were set as follows: (a) the dataset must contain healthy controls, mild, moderate and SA patients; (b) the samples in the dataset must be derived from airway epithelium.
Replication	We used a series of bioinformatics methods to screen for 10 targets related to the severity of asthma and neutrophil infiltration, and verified these targets at the transcriptional level in vivo mouse model and vitro 16HBE cells.
Randomization	We selected a gene expression dataset from the Gene Expression Omnibus (GEO, https://www.ncbi.nlm.nih.gov/geo/) database of the National Center for Biotechnology Information. The selection criteria for the dataset were set as follows: (a) the dataset must contain healthy controls, mild, moderate and SA patients; (b) the samples in the dataset must be derived from airway epithelium.
Blinding	We utilized publicly available data in our study, which makes blinding not applicable. Although blinding can reduce subjective bias in experimental results, it is not feasible under these circumstances. We ensured the validity and reliability of our results by using rigorous statistical methods and following established guidelines and protocols.

Reporting for specific materials, systems and methods

We require information from authors about some types of materials, experimental systems and methods used in many studies. Here, indicate whether each material, system or method listed is relevant to your study. If you are not sure if a list item applies to your research, read the appropriate section before selecting a response.

Materials & experimental systems

n/a	Involved in the study	
☒	☐	Antibodies
☐	☒	Eukaryotic cell lines
☒	☐	Palaeontology and archaeology
☐	☒	Animals and other organisms
☒	☐	Clinical data
☒	☐	Dual use research of concern
☒	☐	Plants

Methods

n/a	Involved in the study	
☒	☐	ChIP-seq
☒	☐	Flow cytometry
☒	☐	MRI-based neuroimaging

Eukaryotic cell lines

Policy information about cell lines and Sex and Gender in Research

Cell line source(s)	Human bronchial epithelial cells (16HBE, HBE135-E6E7) were obtained from Fuhen Biologicals
Authentication	The cell identification certificate for 16HBE was provided by Fuhen Biologicals
Mycoplasma contamination	the cell lines were not tested for Mycoplasma contamination
Commonly misidentified lines (See ICLAC register)	no

Animals and other research organisms

Policy information about studies involving animals; ARRIVE guidelines recommended for reporting animal research, and Sex and Gender in Research

Laboratory animals	BALB/C mice (female, 6-8 weeks old) were obtained from Zhejiang Weitong Lihua Laboratory Animal Technology Co., Ltd. (license number: SCXK (Zhejiang) 2019-0001, Guangzhou, China)
Wild animals	BALB/C mice (female, 42-48 days old) were obtained from Zhejiang Weitong Lihua Laboratory Animal Technology Co., Ltd. (license number: SCXK (Zhejiang) 2019-0001, Guangzhou, China). After the final nebulization, we euthanized the 24 mice and collected their blood, bronchoalveolar lavage fluid, and lung tissue for further experiments.
Reporting on sex	Asthma is a chronic respiratory disease that affects the airways of the lungs, causing inflammation and narrowing of the air passages. It is known to affect humans and animals alike, including mice. In laboratory studies, it has been observed that female mice are more susceptible to developing asthma-related symptoms than male mice. Therefore, we chose to use female mice in our study to better understand the mechanisms and potential treatments for this condition. By studying the effects of different interventions on female mice, we hope to gain insights into the underlying causes of asthma and develop effective therapies for both human patients and animal models.
Field-collected samples	this study did not involve samples collected from the field.
Ethics oversight	This study was approved by the Laboratory Animal Ethics Committee of Guangzhou University of Chinese Medicine (Approval No. 20230413001).

Note that full information on the approval of the study protocol must also be provided in the manuscript.
